# Rule–based regulatory and metabolic model for Quorum sensing in *P. aeruginosa*

**DOI:** 10.1186/1752-0509-7-81

**Published:** 2013-08-21

**Authors:** Nadine S Schaadt, Anke Steinbach, Rolf W Hartmann, Volkhard Helms

**Affiliations:** 1 Center for Bioinformatics, Saarland University, Campus E2.1, 66123 Saarbrücken, Germany; 2 Campus C2.3, 66123 Saarbrücken, Germany; 3 Pharmaceutical and Medicinal Chemistry, Saarland University, Campus C2.3, 66123 Saarbrücken, Germany

**Keywords:** Quorum sensing, Multi–level logical approach, Boolean network, Gene–regulatory network, Inhibitor, *Pseudomonas aeruginosa*, *pqs* system

## Abstract

**Background:**

In the pathogen *P. aeruginosa*, the formation of virulence factors is regulated via Quorum sensing signaling pathways. Due to the increasing number of strains that are resistant to antibiotics, there is a high interest to develop novel antiinfectives. In the combat of resistant bacteria, selective blockade of the bacterial cell–to–cell communication (Quorum sensing) has gained special interest as anti–virulence strategy. Here, we modeled the *las*, *rhl*, and *pqs* Quorum sensing systems by a multi–level logical approach to analyze how enzyme inhibitors and receptor antagonists effect the formation of autoinducers and virulence factors.

**Results:**

Our rule–based simulations fulfill the behavior expected from literature considering the external level of autoinducers. In the presence of PqsBCD inhibitors, the external HHQ and PQS levels are indeed clearly reduced. The magnitude of this effect strongly depends on the inhibition level. However, it seems that the pyocyanin pathway is incomplete.

**Conclusions:**

To match experimental observations we suggest a modified network topology in which PqsE and PqsR acts as receptors and an autoinducer as ligand that up–regulate pyocyanin in a concerted manner. While the PQS biosynthesis is more appropriate as target to inhibit the HHQ and PQS formation, blocking the receptor PqsR that regulates the biosynthesis reduces the pyocyanin level stronger.

## Background

Quorum sensing (QS) describes how the communication between bacteria is established. Thus, the regulation of genes is adapted to cell population density through the activity of a combined regulatory and metabolic network. In *P. aeruginosa*, QS is involved in the formation of biofilms and in the production of a large number of virulence factors, such as elastase, rhamnolipids, and pyocyanin. These virulence factors are responsible e.g. for tissue damage and disruption of tight junctions. *P. aeruginosa* usually infects patients with immune system deficiencies. Since an increasing number of infecting strains are resistant to most current antibiotics, there is a large interest in developing novel antibacterial strategies. It has been suggested that selectively targeting the QS machinery by signaling molecule inhibitors may be advantageous over antibiotics that target central metabolism or DNA replication with respect to the development of resistance mutations because the former strategies have no impact on bacterial viability delay [1,2].

**Figure 1:**
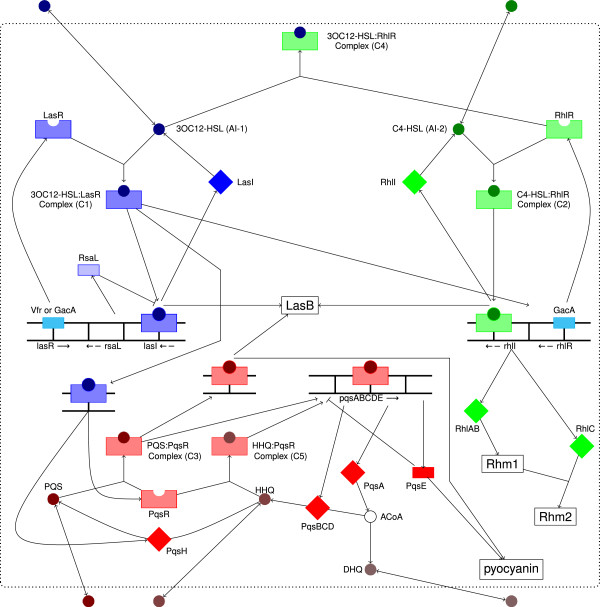
**QS network of *P.aeruginosa*. **Regulation of the virulence factors elastase (LasB), rhamnolipids (Rhm2), and pyocyanin by the hierarchically organized systems *las* (blue), *rhl* (green), and *pqs* (red). Colored balls represent signaling molecules, squares denote enzymes, and colored rectangles are symbols for receptors or other proteins.

Figure 1 gives an overview of the QS of *P. aeruginosa*[3-6]. The QS network consists of three systems termed *las*, *rhl*, and *pqs* that are organized hierarchically (references for the individual reactions are given in Additional file 1: Table S1 and Additional file 2: Table S2). In the *las* system (colored in blue), the synthase LasI is responsible for the biosynthesis of the autoinducer *N*–3–oxododecanoyl–homoserine lactone (AI–1) that binds to the receptor LasR. This AI–1:LasR complex (C1) up–regulates the transcription of LasI as well as of RsaL that blocks the transcription of LasI [7-9]. Furthermore, the *las* system initiates both other QS systems. Likewise, the *rhl* system (colored in green) contains a positive feedback loop that leads to a rapid increase of autoinducer concentration involving the second autoinducer *N*–butyryl–homoserine lactone (AI–2), the receptor RhlR, their complex AI–2:RhlR (C2), and the synthase RhlI [10]. Whereas AI–1 is able to bind to RhlR (complex C4), AI–2 does not bind to LasR [11]. The global activator GacA up–regulates the receptors LasR and RhlR of the first and second QS system, whereas the activator Vfr only up–regulates LasR [12,13]. Finally, the *rhl* system activates the transcription of RhlAB and RhlC that are required to form rhamnolipids [14-16].

The *pqs* system (in Figure 1 colored in red) uses the *Pseudomonas* quinolone signal (PQS) that is synthesized from HHQ by the enzyme PqsH. Both HHQ and PQS are able to form complexes with the receptor PqsR (in the following denoted as C5 and C3) that regulate many genes, such as the biosynthesis operon *pqsA–E*[17,18]. As first step, the enzyme PqsA produces anthraniloyl–CoA using anthranilic acid. Then, PqsD forms HHQ from ACoA and *β*–ketodecanoic acid or its bioactivated thioesters [19]. The enzymes PqsB and PqsC are also required to synthesize HHQ whereby their rule is not completely understood [20,21]. Finally, PqsE acts as negative regulator of the *pqsA–E* operon [20]. In this study, we do not include further regulators related to the QS machinery. For example, it was shown that QscR represses the transcription of *lasI*[22] and VqsR negatively regulates the expression of *qscR*[23].

The dynamic behavior of cellular regulatory networks can be analyzed with a wide range of theoretical approaches: Boolean networks, Kauffman networks, probabilistic Boolean networks, dynamic Bayesian networks, Petri Nets, as well as differential equation models [24-26]. Differential equation models have the advantage of enabling a quantitative analysis. However, for many biochemical reactions the actual rate constants are unknown. Therefore, a Boolean model was used, e.g. to understand the FA/BRCA pathway [27]. A generalized logical formalism with multi–levels was applied to the Gap–gene System in *Drosophila*[28] and to analyze the cell cycle of budding yeast [29].

The dynamic behavior of the QS systems was modeled so far either as simple feedback loop or considering the *Pseudomonaslas* and *rhl* systems using ordinary as well as partial differential equations [30,31] or regarding the *lux* system of *Vibrio fischeri* applying so–called P systems [32]. Anguige *et al.* included a LasR degradation drug in their differential equation approach of the *las* system [33]. Furthermore, the development of biofilms was studied using the *las* system [34] or a 3D growth model of a self–producing signaling molecule including inhibition [35].

In this work, we implemented a multi–level logical approach and compared the influence of enzyme inhibitors and that of receptor antagonists on the formation of autoinducers and virulence factors. Here, different levels of inhibition were considered. Additionally, we analyzed the topology of the network. For this purpose, we modeled the QS in *P. aeruginosa* comprising the *las*, *rhl*, and *pqs* systems as well as the virulence factors elastase, rhamnolipids, and pyocyanin [36,37].

## Methods

**Figure 2:**
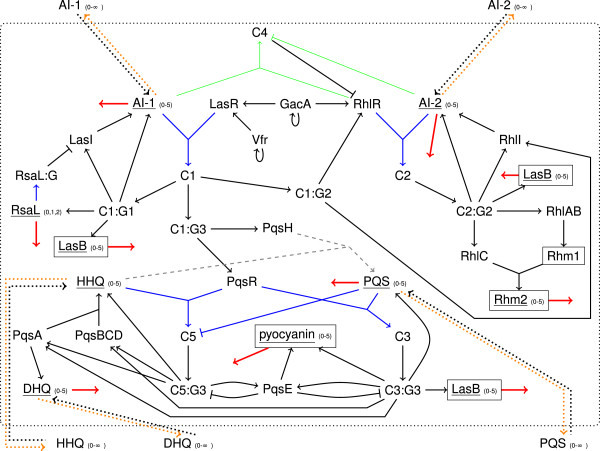
**QS network as a Boolean topology.** Black edge = threshold is 1; blue edge = state of underlined node must be at least 2; orange edge = state of underlined node must be at least 3; green and thin edge = state of underlined node must be at least 4; numbers denote possible states for a node; dotted arrows are reactions involved in a transport process; red and thick edge = happens after a certain number of time steps (degradation). Gray and dashed arrows denote reactions that occurs by chance with a certain probability. Nodes named C represent a complex between autoinducer and receptor, C:G is the complex bound to an operon.

We aimed to adopt a robust formalism that is as independent of parameters as much as possible and that generates easily interpretable results. Since a pure Boolean model is a drastic simplification that does not allow to realize the three hierarchical layered QS systems, we implemented a logical model with multi–level variables. Figure 1 illustrates the connectivity of the three QS systems as a pathway diagram, and Figure 2 shows the same network in a topology suitable for generalized Boolean networks.

### Computational analysis

**Figure 3:**
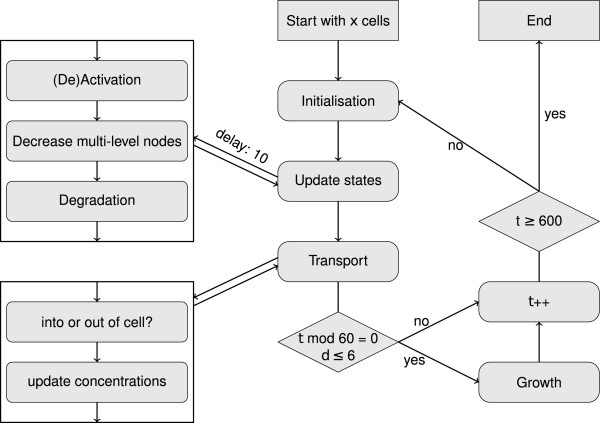
**Flow chart.** Iteration over updating of states, transport, and at certain time points growth (represented as generation of new cells). The updating of states is performed only for cells with a life time longer than a delay time of ten. Here, *t* denotes the time step and *d* the number of cell divisions.

In this multi–level formalism each node can adopt several possible states (see Additional file 3: Table S3). An overview of the updating scheme and work flow is given in Figure 3. We consider a certain number of cells that share the same environment, which means that the external autoinducer concentrations are equal to each cell. Usually, the simulation starts with a single cell. In the results section, we only discuss the results for this first cell. During exponential growth, i.e. before completing six cell divisions, a cell divides into two cells after every 60 time steps. The simulations are iterated until time step 600. Each new cell is initialized in the same way as the first cell and has a delay time of ten steps during which only transport processes are possible. Thus, in this model, the first cell represents a biological cell at the end of the exponential growth phase.

In a single cell, the states of each node are updated according to a synchronous scheme. This means that at each time step, first the states of all (including Boolean and multi–level) nodes are activated or deactivated according to logical analysis considering all connected edges and the states of the attached nodes. The new state *x* of node *i* at the next time step *t*+1 is then determined according to equation (1),

(1) $$x_{i}(t+1)\;=\;M\left(\;H\left(\;\left(\overset{N}{\underset{j=1}{\sum }}w_{\text{ij}}K(x_{j}(t),\varepsilon_{\text{ij}})\right)\;\right)\;+\;G(x_{i}(t),i),i\right)$$

whereby **W**=(*w*_*ij*_) is a weighting matrix and contains all relations between the nodes. Its entry *w*_*ij*_ is $\frac{1}{l}$ when node *j* is one of *l* nodes that are together required to activate node *i*. If node *j* deactivates node *i* then *w*_*ij*_ is −*k* where *k* is the number of reactions that activate node *i*. Otherwise, *w*_*ij*_ is zero. In contrast to some other Boolean models, the second case implies that one inhibiting reaction overrules the activating reactions of node *i*. Also, a node that is not explicitly activated at a certain time step is inactive. As our network only included deactivating nodes in cases where there is a good evidence that they inhibit certain activations, the weighting scheme makes sure that deactivations are stronger than activations. As the concentration of the autoinducers must reach a certain threshold *ε* to start the positive feedback loop, some reactions in our model are only activated if a critical threshold of certain metabolites is exceeded. Function *K* controls that the state of every node *j* that is related to node *i* has reached the required threshold at time *t*. Function *H* rounds the weighted sum to an integer valued output. For multi–level nodes with more than two possible states, i.e. autoinducers, RsaL, and virulence factors the level at the current time step *x*_*i*_(*t*) is added via function *G*. Function *M* ensures that no node adopts a higher value than its maximum possible value. The explicit forms of the functions *M*, *H*, *K*, and *G* are given in Additional file 4.

After updating all nodes, the states of multi–level nodes are decreased by the number of reactions in which they were used. Let us assume, that for example, HHQ forms a complex with PqsR and HHQ is additionally required to yield PQS. Then, in this step the level of HHQ is decreased by two. Those multi–level nodes are subject to a further reaction that decreases the state value after every 20 time steps to account for degradation (see Section Choice of parameters). This degradation is realized equally for all multi–level nodes independent of their maximum possible states. A time step represents either the whole formation of a protein including transcription and translation or a faster enzymatic reaction. Hence, a degradation happens infrequently. However, a node has a relatively small number of possible states, such that a single degradation process consumes a comparably large part of its concentration. For example, in the case of RsaL a degradation decreases the level by two–thirds. Transport reactions (dotted lines in Figure 2) are neither considered in the logical updating scheme nor in the decreasing step afterwards. Additionally, we included some random reactions for processes that take place either infrequently or very slowly in comparison to the other processes, for example, the transformation of HHQ to PQS [18]. For this, we used a random reaction that happens about every fourth step. For simplicity, the enzymes PqsB, PqsC, and PqsD are considered as one node.

The communication between different cells is based on the exchange of the signaling molecules. For this, we modeled a simple transport process that occurs after updating all states inside the cells. When the internal concentration of an autoinducer is higher than the transport threshold, it is transported out of the cell except for the case where the external concentration relative to the number of cells is higher than the internal concentration. Otherwise, for external levels larger than zero, an autoinducer is transported into the cell. In the case that there is no autoinducer outside, there is also no transport. We do not distinguish between diffusion and efflux pumps or active transports and apply usually a transport threshold of three, i.e. if the autoinducer concentration is high.

#### Knock–out mutants, enzyme inhibitors and receptor antagonists

**Figure 4:**
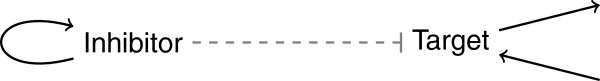
**Realization of inhibitors.** The inhibitor that is initialized as being active blocks its target with a certain probability. The time point when the dashed and gray colored reaction happens is realized randomly.

Besides the wild type also knock–out mutants and inhibitors were considered. For such mutants (PqsR ^−^, PqsA ^−^–PqsBCD ^−^, PqsE ^−^, PqsE ^−^–PqsR ^−^, PqsE ^−^–PqsA ^−^–PqsBCD ^−^) all respective formation edges were removed. Conversely, inhibitors (PqsR antagonist, PqsBCD inhibitor) block a certain fraction of the target. Therefore, inhibition of the target was set to happen with a certain inhibition level that indicates at what percentage the target is blocked (see Figure 4). An inhibitor is initialized as being active, but the time when the inhibitor blocks its target is chosen randomly.

## Result and discussion

We constructed a combined regulatory and metabolic network for the three QS systems in *P. aeruginosa*. The realization as a dynamic rule–based computational model also enabled us to study the effects of gene knock–outs and those of enzyme inhibitors as well as receptor antagonists.

### Choice of parameters

For the sake of simplicity and interpretability, the model was designed as a minimal system. A synchronous, parameter–free Boolean logical analysis was applied where nodes can be either “on” or “off” except for some nodes and edges that are discussed below in detail. In a Boolean network with a synchronous updating scheme, there is no time scale so that all reactions take place at the same time with the same rate. This is a valid approach unless this alters the system behavior in a crucial waycompared to either considering different reaction time scales, or if certain reactions depend on special conditions. We will show in the following that the first assumption (same time scale) is not crucial for the system properties studied here. The second point, however, required a generalization of the Boolean model by introducing multi-level logical functions at various points of our model.

We will now discuss the elements of the systems where more than two states were found necessary. (i) The regulator RsaL blocks the transcription of the autoinducer synthase LasI. Hence, three different RsaL levels were used, namely “not available” (0), “available at low concentrations” (1), and “sufficient to block LasI” (2) are used. (ii) Besides PQS, HHQ is also able to bind to PqsR, and to activate PqsR, but PQS has a 100–fold higher affinity than HHQ [18]. Therefore, PQS was set to inactivate the complex C5 of HHQ and PqsR with a threshold of two representing a large concentration of PQS. (In Figure 2, all reactions with a threshold of two are colored in blue). (iii) Since the activation of gene expression (activation of Ci:Gi by Ci) is concentration–dependent, the complexation between autoinducer and receptor occurs only when the autoinducer concentration has reached the lower threshold of two. (iv) Moreover, the autoinducer of the *las* system AI–1 is able to form a weak complex with the second receptor RhlR as long as the autoinducer of the *rhl* system AI–2 has not reached a very high concentration. Thus, formation of the complex C4 between AI–1 and RhlR required at least four AI–1 and its inactivation at least four AI–2 (edges colored in green in Figure 2). (v) Due to the accumulation of autoinducers and virulence factors, six different levels were considered for the internal signaling molecules AI–1, AI–2, HHQ, as well as PQS and the virulence factors. The external autoinducer levels were allowed to adopt arbitrarily large numbers.

#### Conversion frequency

**Figure 5:**
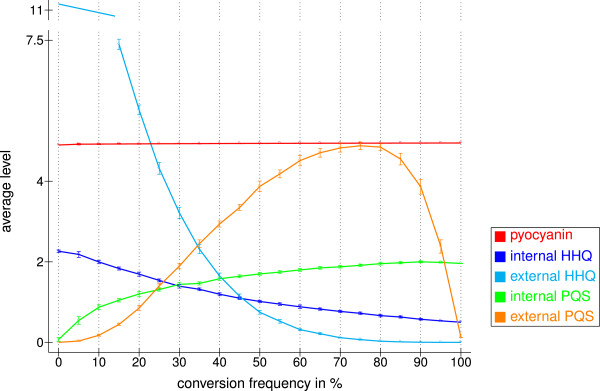
**Effect of the PQS production rate.** Effect on the average levels of the autoinducers HHQ and PQS, as well as that of pyocyanin. The reported values are mean levels in the time interval 100 to 600 averaged over ten runs obtained with different random numbers and their standard deviations. The conversion frequency represents the probability that PQS is produced by PqsH. The degradation frequency was set to once per 20 time steps and the transport threshold to three.

HHQ is formed by one cell, transported into another cell, and there converted into PQS [17]. Although this may yield to a time delay in reality, our model assumes that both steps take place in a single cell. Therefore, we designed this reaction (colored in gray in Figure 2) such that it occurs randomly with a certain probability. Figure 5 shows how the autoinducer and pyocyanin levels depend on the reaction rate of this process. Due to the activation of pyocyanin biosynthesis by PqsE and the production of PqsE by the complex C5 using HHQ, the pyocyanin level is independent of the reaction rate. Kesarwani *et al.* reported that the HHQ concentration is about 12% of the PQS concentration in the beginning of the stationary growth phase [38]. To match this experimental finding, we used a conversion frequency of 55% in the following.

#### Degradation

If no degradation steps were included, all metabolites would be present for the whole time after their production. Hence, the state levels of the virulence factors would be at maximal levels since they have no other out–going edges. This is not realistic because metabolites are generally degraded after a certain time in a cell or may be consumed by reactions not considered in our network. Therefore, we have also considered degradation steps in our model that occur after the states have been updated according to the producing reactions. After a degradation step, no node reaches its maximum level anymore. We found that if degradation takes place after every time step, the three QS systems become inactive except for the proteins Vfr and LasR due to the self–loop of Vfr and the relation between them and the applied initialization conditions.

Additional file 5: Figure S1 shows how different degradation frequencies between 1 and $\frac{1}{50}$ influence the levels of virulence factors. If degradation occurs after five or more time steps the degradation frequency generally has only a very small effect. We compared the virulence factor level with the theoretical possible maximum values considering the numbers of degradations in the simulation time considered. The smaller the frequency the larger is the effect of the autoinducer degradation on the virulence factor level. The pyocyanin level comes close to the maximal level for frequencies of $\frac{1}{7}$ or less and reaches this value at a degradation frequency of $\frac{1}{20}$ or less. Therefore, we chose a degradation frequency of $\frac{1}{20}$ for all following computations.

#### Transport threshold

In our model, transport is subject to concentration gradients between individual cells and the exterior. For transport out of a cell to occur, the autoinducer level must also overcome a certain threshold. Additional file 6: Figure S2 shows the influence of this threshold on the level of virulence factors. In general, the effect of different threshold values between 1 and 5 is negligible, in particular when considering the time interval from 100 to 600. As expected, the external autoinducer levels decrease with an increasing transport threshold, whereas the internal levels increase. For thresholds larger or equal to four, the levels of external HHQ and PQS are zero. If the transport threshold is set to one, the internal HHQ and PQS levels are so small that the pyocyanin level is slightly decreased. In the following, we used a transport threshold of three, which corresponds to a relatively high autoinducer concentration.

### Initialization and starting behavior

In this study, we are primarily interested in identifying attractors in which the QS systems are active. Obviously, when all initial states of the network are set to zero (inactive or not available), the whole system will remain inactive. The *las* system becomes active when (1) one or both of the global activators GacA and Vfr are initially set to one and when (2) the complex C1 between AI-1 and LasR binds to the *las* operon (C1:G1 is set to one). The *rhl* system is activated with a certain time delay. This delay can be avoided when GacA, C1:G1, and additionally C2:G2 (complex C2 between AI–2 and RhlR binds to the *rhl* operon) are active in the beginning. If in the beginning C5:G3 and C3:G3 are activated as well, the *pqs* system becomes active after a short delay, too. Also, switching on PqsR avoids the delay. In general, the initialization of Cx:Gy (complex Cx bound to operon Gy) to one is necessary since basal transcription rates are not included in the logical approach used here. In our system, the basal transcriptions could be included either with the same rate as the activated gene expression or in a stochastic way as often as degradation. The second case does not result in an initiation of the system. Alternatively, it is possible to start with elevated autoinducer levels or to begin with externally available autoinducers that are able to diffuse or can be transported into the cells.

When more nodes are initially active, the initialization phase of the system takes a different route because it is no longer required to start up the whole system. However, at later times, the behavior and the levels are the same as using minimal initial conditions. Additional file 7: Figure S3 shows that, after about 100 time steps, the metabolite levels reached in a computation with all nodes initially activated are very similar to those reached with minimal initial conditions. For all other further considerations, we used minimal initial conditions in which only Vfr, C1:G1, C3:G3, and C5:G3 are initially set to one.

Additional file 8: Table S4 shows an example trajectory where the system has reached a stable regime after time 100. Therefore, we can consider all time steps after 100 for computing equilibrated average values. For statistical reasons, we carried out all simulations until a time of 600. The differences of the levels using different growth rates were found to be very small. We compared growth processes using different division frequencies at time steps 60, 120, and 240 and modified the simulation time such that in each case about 200 time steps in stationary phase are possible. For most internal nodes, the levels were the same. Due to the stochastically modeled formation of PQS, the levels differ from each other in the same range as between different runs that used the same growth parameters but different random numbers for the *pqs* system. The external levels also display only negligible variations due to degradations. For example, the external level of autoinducer AI–1 divided by the total number of cells is 3.1 for a growth rate of 60, 3.3 for a growth rate of 120, and 2.9 for a growth rate of 240 averaged over the last 100 time steps. When comparing the behavior of a single cell over 600 time steps to that of 64 cells the internal and external levels are slightly changed. However, the qualitative behavior remains unchanged as well. For a single cell, the external level of AI–1 averaged over the last 100 time steps is 2.6. Hence, the behavior of the model is practically independent of the growth processes. In the following, we used a growth rate of 60 time steps and 6 division processes.

**Figure 6:**
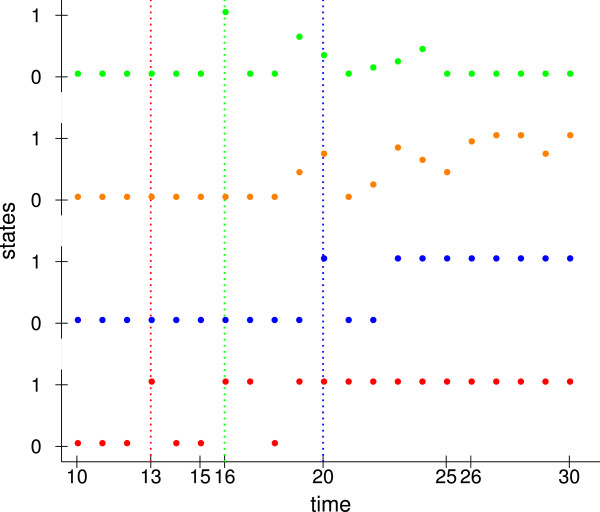
**Start behavior.** Based on the complexes using minimal initial conditions. The first complex C1 between AI–1 and LasR is shown in red and the second complex C2 between AI–2 and RhlR in blue. The levels averaged over ten different runs for complexes of the *pqs* system are shown in orange (C3 between PQS and PqsR) and in green (C5 between HHQ and PqsR). Dotted lines denote the first peak of the same colored complex.

Figure 6 shows the behavior of the complexes at the start of the simulation. In general, we defined a delay time of 10 time steps for each new cell during which only transport processes are possible. At first, the *las* system becomes active. The first peak is at time 13 because three reactions are required to build complex C1 starting from C1:G1. Those reactions include the whole biosynthesis. After time 19, the *las* complex C1 is always active. After a short delay, the *rhl* and *pqs* systems are activated since C1 is necessary to activate C1:G2 and C1:G3. Three further reactions are required to build the *rhl* complex C2. However, C2 is not activated directly since the level of AI–2 is too low. Therefore, these reactions have to take place twice. Then, the first peak of C2 is at time 20. The first peak of the C3 and C5 complexes of the *pqs* system occurred in the time interval between 19 to 23 and at time 16. PqsR and PqsH are activated by C1:G3 at time 15 for the first time. Until time 15 the level of HHQ is two and the level of PQS is one, such that at time 16 the complex C5 between PqsR and HHQ is formed. Afterwards, the activation of C3 and C5 depends on the stochastically modeled formation of HHQ by PqsH. When C3 is active C5 is inactive since PQS inhibits C5.

### Behavior of wild type and knock–out mutants

**Figure 7:**
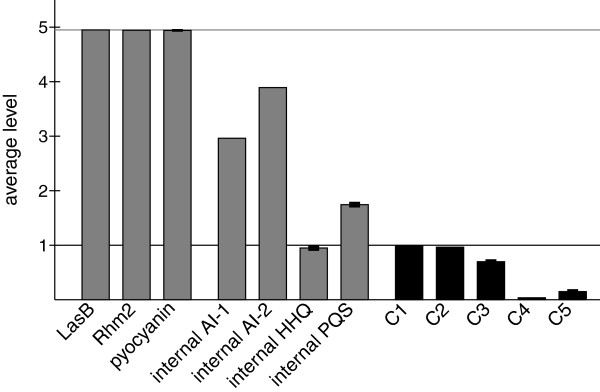
**Behavior of wild type.** Lines denote the maximum value for bars with the same color. Shown are average levels in the time interval 100 to 600 averaged over ten runs obtained with different random numbers and their standard deviations.

In the following, network initialization is performed as before using the described minimal system. Figure 7 summarizes the behavior of the wild type system after the starting phase. The virulence factors LasB, Rhm2, and pyocyanin are generated at maximum level. The level of AI–2 is higher than the levels of AI–1, HHQ, and PQS since there is no negative regulator in the *rhl* system whereas there is RsaL in the *las* system and PqsE in the *pqs* system. Due to the high level of AI–2, the complex C4 between the first autoinducer AI–1 and the second receptor RhlR is inhibited. Complex C5 that is formed from PqsR and HHQ is clearly less active than the complex C3 between PqsR and PQS since the averaged internal PQS level is almost two.

**Figure 8:**
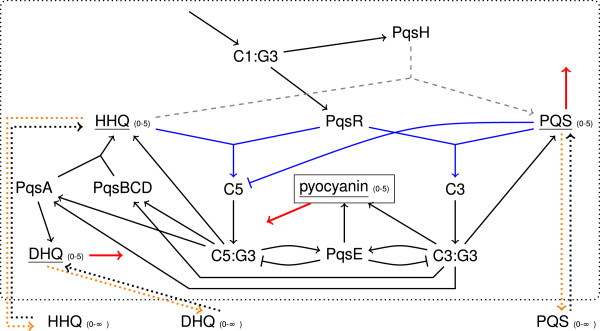
**Detailed view of the *pqs *system.** Black edge = threshold is 1; blue edge = state of underlined node must be at least 2; orange edge = state of underlined node must be at least 3; numbers denote the number of possible states for a node; dotted arrows are reactions involved in a transport process; red and thick edge = happens after 20 time steps (degradation). gray and dashed edge denotes a reaction that occurs by chance with a certain probability.

Now, we focused on the *pqs* system shown in Figure 8 in order to illustrate the behavior of the network topology in detail. All other parts of the network were still initialized and processed as described before. Additionally, we considered the effects of knock–out mutants and inhibitors of the PQS biosynthesis or antagonists of the receptor PqsR. As mentioned before, the complexation between autoinducer and receptor requires at least an autoinducer level of two in our model (reactions colored in blue). Further, when the level of PQS is two or higher, HHQ can no longer bind to PqsR (inhibition of C5 by PQS colored in blue). HHQ and PQS can only be transported outside of the cell when their level is three or higher (colored in orange). The red colored edges denote degradations that decrease the level of the respective node by one after every 20 time steps. The gray colored reaction happens stochastically with a conversion frequency of 55%.

**Figure 9:**
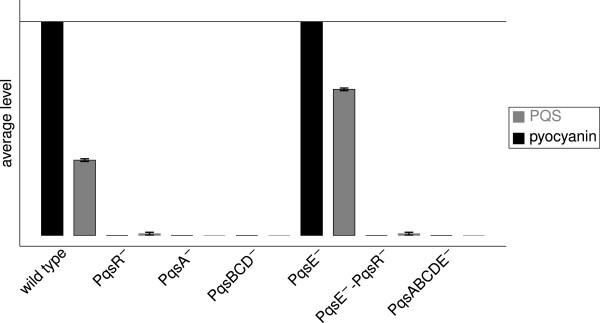
**Calculated PQS and pyocyanin levels for wild type and knock–out mutants.** Displayed are average values in the time interval 100 to 600 averaged over ten runs obtained with different random numbers and their standard deviations.

Figure 9 shows the behavior of the knock–out mutants modeled here, namely PqsR ^−^, PqsA ^−^, PqsBCD ^−^, and PqsE ^−^, as well as the double mutants PqsA ^−^– PqsBCD ^−^, PqsE ^−^–PqsR ^−^ and PqsE ^−^–PqsA ^−^–PqsBCD ^−^ (PqsABCDE ^−^) in comparison to the wild type. Knock–out mutants within the *pqsA–E* operon and *pqsR* are known to produce zero or drastically reduced amounts of pyocyanin compared to wild type cells [17,20,39]. As reported in the literature deleting PqsR, PqsA, or PqsBCD in our model results in a deficiency in PQS and pyocyanin formation. The single PqsE ^−^ knock–out mutant reached a higher internal PQS level than the wild type due to the missing inhibition of C3:G3 and C5:G3 by PqsE and therefore produced pyocyanin near the maximum level. Yet, it was reported that PqsE ^−^ knock–out mutants produce clearly less pyocyanin than wild type cells whereas an increased concentration of PqsE enhances the pyocyanin concentration [20,40]. We will return to this issue below where we will investigate the network topology around PqsE in detail. PqsE is able to regulate pyocyanin independent of PQS and PqsR, but subject to the *las* and *rhl* system [40]. The external level of PQS in a PqsE ^−^ mutant is strongly increased. In contrast, the external concentration of autoinducers was reported to be similar in the wild type and PqsE ^−^ knock–out mutants [40]. However, in PqsE ^+^ mutants the concentration of HHQ and PQS is reduced [20].

### Behavior of enzyme inhibitors and receptor antagonists

**Figure 10:**
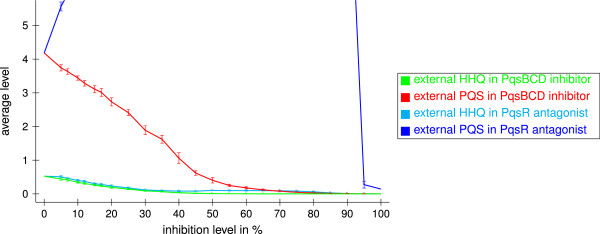
**External HHQ and PQS levels of wild type and inhibitors with varying inhibition levels.** Shown are average values in the time interval from 100 to 600 averaged over ten runs obtained with different random numbers and their standard deviations.

Next, we analyzed the effect of blocking different target proteins on the levels of the autoinducers HHQ and PQS as well as the level of the virulence factor pyocyanin. We compared inhibitors of the signal molecule biosynthesis and antagonists of the PqsR receptor and studied the influence of the inhibition level. For example, an inhibition level of 40% means that 40% of all receptors or enzymes of the corresponding type are blocked. Figure 10 shows the external HHQ and PQS levels for the unblocked system (inhibition level of 0%) and for systems including inhibitors.

**Figure 11:**
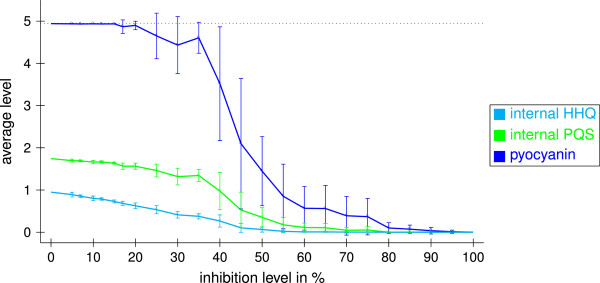
**Enzyme inhibition.** Inhibition of PqsBCD with varying inhibition levels. Shown are average values in the time interval from 100 to 600 averaged over ten runs obtained with different random numbers with and standard deviations. The dotted line represents the theoretical maximum possible values.

In the presence of PqsBCD inhibitors, the external HHQ and PQS levels are noticeably decreased with a high dependence of the inhibition level. For example, a PqsBCD inhibitor with inhibition level of 30% reduces the external HHQ level by about 80% and the external PQS level by about 55% (see Figure 10). Indeed, Storz and coworker showed that a PqsD inhibitor reducing the HHQ concentration by 77% also reduced the PQS concentration by 42% [41]. Figure 11 shows the effect of PqsBCD inhibitors with varying inhibition levels on the internal autoinducer and pyocyanin levels. The pyocyanin level is also clearly decreased. For PqsBCD inhibitors with an inhibition level of 30%, it is reduced by about 10%.

**Figure 12:**
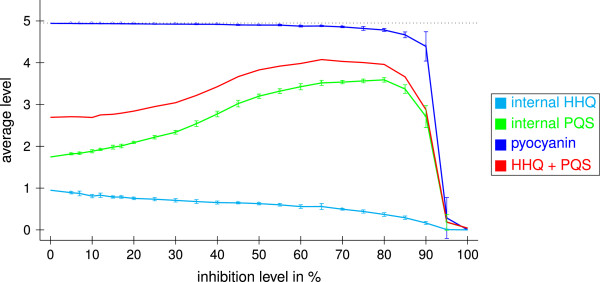
**Receptor antagonists.** Inhibition of PqsR with varying inhibition levels. Shown are average values in the time interval from 100 to 600 averaged over ten runs obtained with different random numbers and their standard deviations. The dotted line represents the theoretical maximum possible values.

The external PQS level is strongly increased by PqsR antagonists due to missing usage for complex C5 (see Figure 10). Figure 12 shows the effect of weak and strong PqsR receptor antagonists on the internal HHQ and PQS levels and on the pyocyanin level. As long as less than 60% of PqsR is blocked, the pyocyanin level is only very slightly decreased. In contrast, Klein *et al.* presented PqsR antagonists with affinity in the low micromolar range that indeed reduce the pyocyanin concentration (IC_50_: 87 *μ*M) [42]. Further, it was reported that PqsR antagonists, derivates of the natural effector HHQ, with K _*D*_ values in a low nanomolar range reduce the pyocyanin formation by about 75% at 3 *μ*M [43].

### Updated network

While the behavior of the HHQ and PQS levels mostly fulfills experimental observations, the discrepancy for pyocyanin between predicted and observed behavior suggests that pyocyanin production is co–regulated by further functionally unknown proteins that may or may not be connected to the *pqs* system. Here, we only considered the connected case. Thus, we proceeded by studying networks with modified connectivities. For simplicity, we did not consider negative regulations or varying conditions such as reaction rates or modifications in the number of states for certain nodes. Instead of the feed–forward link *pqs*–member → X → pyocyanin we used a direct link from *pqs*–member → pyocyanin such that we do not have to include new hypothetical proteins. We further assumed that relations are only possible between a protein acting as receptor and a corresponding signaling molecule.

Given these assumptions Table 1 shows all theoretically possible reactions to form pyocyanin whereby pyocyanin is regulated either via PqsE and therefore by PqsR (labeled as underlined) or via PqsA, PqsBCD, and therefore PqsR (as bold). Reactions that share both conditions are labeled in italic. In order to match the behavior of our computational model to the existing knowledge about pyocyanin formation (PqsR, PqsA, PqsBCD, and PqsE are required), we either have to combine a reaction labeled as bold with a reaction labeled as underlined or take an italic labeled reaction. Instead of a bold labeled reaction, we can also use the reaction pairs R6 and R7, R14 and R7, R11 and R7, or R8 and R7, respectively. R20 together with R7 fulfills “italic” conditions. Since PqsH is not influenced by mutants or inhibitors considered here, we did not consider reactions involving PqsH (R5 and R9–R11). PqsA and PqsD are involved in the formation of HHQ. Hence, we also did not use reactions R12–R17. To increase the effect of PqsR antagonists on the pyocyanin formation either R4 has to be added to possible combinations of reactions or reactions R21–R24 have to be considered.

**Table 1:** Possible reactions to form pyocyanin

**Notation**	**Reaction**
R1	PqsE → pyocyanin
**R2**	C3:G3 → pyocyanin
**R3**	C5:G3 → pyocyanin
R4	PqsR → pyocyanin
R5	PqsH → pyocyanin
R6	PqsA → pyocyanin
R7	PqsBCD → pyocyanin
R8	PqsR + DHQ → pyocyanin
**R9**	PqsH + HHQ → pyocyanin
**R10**	PqsH + PQS → pyocyanin
R11	PqsH + DHQ → pyocyanin
**R12**	PqsA + HHQ → pyocyanin
**R13**	PqsA + PQS → pyocyanin
R14	PqsA + DHQ → pyocyanin
**R15**	PqsBCD + HHQ → pyocyanin
**R16**	PqsBCD + PQS → pyocyanin
**R17**	PqsBCD + DHQ → pyocyanin
*R18*	PqsE + HHQ → pyocyanin
*R19*	PqsE + PQS → pyocyanin
R20	PqsE + DHQ → pyocyanin
*R21*	PqsR + PqsE + HHQ → pyocyanin
*R22*	PqsR + PqsE + PQS → pyocyanin
R23	PqsR + PqsE + DHQ → pyocyanin
R24	PqsR + PqsE → pyocyanin

Pyocyanin is positively regulated by PqsE (and therefore also PqsR) in reactions labeled as underlined, by PqsA and PqsBCD (and therefore also PqsR) in reactions labeled in bold, as well as by both in reactions labeled in italic.

Table 2 shows all considered networks and their effect on the pyocyanin production. For the networks N1 (discussed in detail in previous sections), N4, N5, N8, N11, and N12, a conversion frequency of 55% reproduces the behavior expected from experiment. In the case of the networks (N2, N3, N6, N7, N9, and N10), where HHQ or PQS are used to form pyocyanin, the probability to convert HHQ into PQS has to be changed. In the following, a conversion frequency of 30% is used for the networks N2, N6, and N9 and 50% for the networks N3, N7, and N10.

**Table 2:** Behavior of updated networks

**Network**	**Used reactions**	**Results**			
		**PqsA**^**−**^**– PqsBCD**^**−**^	**PqsE**^**−**^	**PqsR antagonists**	**PqsBCD inhibitors**
N1	R1, R2	- - - - -	Pyocyanin	Pyocyanin	- - - - -
N2	R18	- - - - -	- - - - -	Pyocyanin	- - - - -
N3	R19	- - - - -	- - - - -	Pyocyanin	PQS
N4	R20, R2	- - - - -	Pyocyanin	Pyocyanin	- - - - -
N5	R1, R2, R4	Pyocyanin	Pyocyanin	Pyocyanin	- - - - -
N6	R18, R4	Pyocyanin	Pyocyanin	Pyocyanin	- - - - -
N7	R19, R4	Pyocyanin	Pyocyanin	Pyocyanin	PQS
N8	R20, R2, R4	Pyocyanin	Pyocyanin	Pyocyanin	- - - - -
N9	R21	- - - - -	- - - - -	- - - - -	- - - - -
N10	R22	- - - - -	- - - - -	- - - - -	PQS
N11	R23, R2	- - - - -	Pyocyanin	Pyocyanin	- - - - -
N12	R24, R2	- - - - -	Pyocyanin	Pyocyanin	- - - - -

Entries denote deviations (too high levels) from the expected behavior.

**Figure 13:**
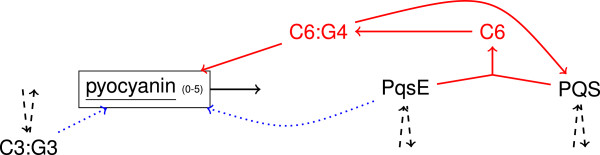
**Modified network termed N3.** Numbers denote possible states for a node; red colored elements denote the number of new nodes or edges, whereas blue dotted lines are edges of the original network removed in this network. Dashed lines represent reactions given in Figure 8.

So far, the natural ligands of PqsE are unknown and it was shown that PQS does not bind to PqsE [44]. According to reaction R19, formation of a complex between PQS and PqsE would lead to up–regulation of pyocyanin. The corresponding modified network is illustrated in Figure 13 and denoted as N3. We postulate that the formation of complex C6 does not require a high concentration of PQS. The edge from C6:G4 to PQS represents the degradation of the complex.

In the networks N5, N6, N7, and N8, PqsA ^−^–PqsBCD ^−^ and PqsE ^−^ mutants produce pyocyanin at maximum level due to the postulated reaction R4, see Table 2. According to this or–linkage, PqsR is essential for the biosynthesis of pyocyanin, so that its level is not decreased in PqsA ^−^–PqsBCD ^−^ and PqsE ^−^ mutants. The levels of all other metabolites are the same as for the corresponding network without reaction R4, e.g. N5 behaves as N1. Besides N1 PqsE ^−^ mutants also produce pyocyanin at maximum level in networks N4, N11, and N12. In the case of N2 and N3, where pyocyanin is activated by a complex between PqsE and HHQ or PQS, no pyocyanin is formed by knock–out mutants. However, weak PqsR antagonists again do not decrease the pyocyanin level.

We consider the networks N9 and N10 as being the closest to literature. In our model, PqsBCD inhibitors are more suitable to reduce the HHQ and PQS level and PqsR antagonists reduce the pyocyanin level at higher rate. In general, the levels strongly decrease with increasing inhibition level and the level of HHQ is clearly stronger decreased than the PQS and pyocyanin levels for the same inhibition level. In the case of network N10, a PqsBCD inhibitor strongly reduces the external HHQ level, whereas the effect on the external PQS level is clearly not as emphasized as for comparable inhibitors of the other networks. In reality, reactions R21 and R22 may be represented in two ways. (1) Two complexes up–regulate the transcription of two different enzymes. The first enzyme catalyzes the formation of a metabolite that is used by the second enzyme to form pyocyanin. (2) A first complex up–regulates the transcription of an enzyme that catalyzes the formation of a certain metabolite and a second complex up–regulates a receptor protein that forms a third complex with this metabolite. Then, the third complex up–regulates the transcription of pyocyanin. In both ways, either the first or the second complex contains PqsR and the other PqsE and one of those receptors uses an autoinducer as substrate. The unknown proteins in our model may be directly or indirectly related to the proteins MvaT and MvaU that are required to form pyocyanin [45]. It was reported that the biosynthesis of pyocyanin is a complex pathway including two operons that share high sequence homology. These are responsible for forming a precursor as well as the enzymes PhzM and PhzS which are required to convert this precursor to pyocyanin [46]. Therefore, we suggest that PqsE might be a regulatory protein and that pyocyanin biosynthesis might be regulated by the two regulators PqsR and PqsE as well as an autoinducer as ligand.

## Conclusions

We have introduced a novel rule–based logical model for QS in *P. aeruginosa*. The model incorporates the key steps of bacterial cells leading to the synthesis of autoinducer molecules and virulence factors. Despite its simplicity, the model enables almost quantitative predictions about the effect of inhibitors of PQS biosynthesis and antagonists of their corresponding receptor PqsR. Also, one can easily investigate the effect of reviewed network topologies. We were able to identify PqsR and PqsE as a key region of the network where the predicted effects of pyocyanin production deviate from experiment. We suggest additional regulatory interactions that can be validated experimentally. In the future, this approach can serve as basis for further integrating the effect of random mutations in various parts of the network.

## Competing interests

The authors declare that they have no competing interests.

## Authors’ contributions

NSS constructed the model and performed calculations. NSS and VH analyzed the results and prepared the manuscript. AS and RWH helped with the biological and pharmaceutical background and edited the manuscript. All authors read and approved the final manuscript.

## Supplementary Material

Additional file 1 **Table S1 References.** References for reactions in QS systems. Click here for file

Additional file 2 **Table S2 References.** References for virulence factor formation. Click here for file

Additional file 3 **Table S3 Multi–level nodes.** Multi–level nodes with their corresponding maximal possible states. Click here for file

Additional file 4 **Equations.** Explicit form of functions required in equation (1). Click here for file

Additional file 5 **Figure S1 Influence of degradation frequency.** Influence on the concentration levels of the three virulence factors. Relative average values are reported in the time interval 100 to 600 averaged over ten runs obtained with different random numbers. Concentrations of 1 denote values equal to the theoretical maximum value. We used a conversion frequency of 55% for converting HHQ into PQS and a transport threshold of three. Click here for file

Additional file 6 **Figure S2 Effect of different transport thresholds on elastase production.** Starting behavior of LasB comparing a threshold of one (box) and six (square). Click here for file

Additional file 7 **Figure S3 Influence of different initializations.** Influence on LasB, pyocyanin, PqsBCD, and PQS: average values in different time intervals relative to theoretical maximum values averaged over ten runs with different random numbers. In the case of min, a minimal set of nodes (Vfr, C1:G1, C3:G3, and C5:G3) is initially activated, while max means that all nodes (except for LasB, Rhm2, pyocyanin, and external autoinducers) were set to one in the beginning. Click here for file

Additional file 8 **Table S4 Example trajectory.** Level values of nodes in the *pqs* system in the time interval 10 to 150 considering a wild type cell of the original network with a minimal initial setup. Click here for file
