# Supplementary material for: Rule–based regulatory and metabolic model for Quorum sensing in P. aeruginosa
Source: BMC Syst Biol. 2013 Aug 21;7:81. doi: 10.1186/1752-0509-7-81 (PMC3765737; doi:10.1186/1752-0509-7-81)
Supplement: Additional file 1 — Table S1 References. References for reactions in QS systems. [file 1752-0509-7-81-S1.pdf]

Reaction	Type	Reference
GacA → LasR	activation, transcription + translation	Reimmann et al. (1997) Mol. Microbiol.
GacA → RhlR	activation, transcription + translation	Reimmann et al. (1997) Mol. Microbiol.
Vfr → LasR	activation, transcription + translation	Albus et al. (1997) J Bacteriol.
AI-1 + LasR → C1	association	Seed et al. (1995) J Bacteriol.
C1 → C1:G1	activation	Seed et al. (1995) J Bacteriol.
C1:G1 → LasI	transcription + translation	Seed et al. (1995) J Bacteriol.
C1:G1 → RsaL	transcription + translation	de Kievit et al. (1999) J Bacteriol.
RsaL → RsaL:G	activation	de Kievit et al. (1999) J Bacteriol.
RsaL:G −| LasI	inhibition	de Kievit et al. (1999) J Bacteriol.
LasI → AI-1	enzymatic reaction (formation)	Passador et al. (1993) Science
AI-1 + RhlR → C4	association	Pesci et al. (1997) J Bacteriol.
C1 → C1:G2	activation	Pesci et al. (1997) J Bacteriol.
C1:G2 → RhlR	transcription + translation	Pesci et al. (1997) J Bacteriol.
AI-2 −| C4	blocking	Pesci et al. (1997) J Bacteriol.
C1:G2 → RhlI	transcription + translation	- - - - -
AI-2 + RhlR → C2	association	Ochsner and Reiser (1995) PNAS
C2 → C2:G2	activation	Ochsner and Reiser (1995) PNAS
C2:G2 → RhlI	transcription + translation	Ochsner and Reiser (1995) PNAS
RhlI → AI-2	enzymatic reaction (formation)	Ochsner and Reiser (1995) PNAS
C1 → C1:G3	activation	Rampioni et al. (2010) Environ. Microbiol.
C1:G3 → PqsR	transcription + translation	Rampioni et al. (2010) Environ. Microbiol.
C1:G3 → PqsH	transcription + translation	Rampioni et al. (2010) Environ. Microbiol.
HHQ + PqsH → PQS	enzymatic reaction (formation)	Deziel et al. (2004) PNAS
PQS + PqsR → C3	association	Deziel et al. (2004) PNAS
C3 → C3:G3	activation	Deziel et al. (2004) PNAS
C3:G3 → PqsABCDE	transcription + translation	Deziel et al. (2004) PNAS
PqsA + PqsBCD → HHQ	enzymatic reaction (formation)	Deziel et al. (2004) PNAS
HHQ + PqsR → C5	association	Xiao et al. (2006) Mol. Microbiol.
C5 → C5:G3	activation	Xiao et al. (2006) Mol. Microbiol.
C5:G3 → PqsABCDE	transcription + translation	Xiao et al. (2006) Mol. Microbiol.
PqsA → DHQ	enzymatic reaction (formation)	
