# Supplementary material for: Rule–based regulatory and metabolic model for Quorum sensing in P. aeruginosa
Source: BMC Syst Biol. 2013 Aug 21;7:81. doi: 10.1186/1752-0509-7-81 (PMC3765737; doi:10.1186/1752-0509-7-81)
Supplement: Additional file 2 — Table S2 References. References for virulence factor formation. [file 1752-0509-7-81-S2.pdf]

Reaction	Reference
C1:G1 → LasB	Gambello and Iglewski (1991) J Bacteriol.
C2:G2 → LasB	Wilder et al. (2011) ISME J
C3:G3 → LasB	Wilder et al. (2011) ISME J
C2:G2 → RhlAB	Ochsner et al. (1994) J Biological Chem.; Medina et al. (2003) J Bacteriol.
C2:G2 → RhlC	
RhlAB → Rhm1	Ochsner et al. (1994) J Biological Chem.; Maier and Soberon-Chavez (2000) Appl Microbiol Biotechnol
Rhm1 + RhlC → Rhm2	Ochsner et al. (1994) J Biological Chem.; Maier and Soberon-Chavez (2000) Appl Microbiol Biotechnol
C3:G3 → pyocyanin	
PqsE → pyocyanin	
