# Supplementary material for: Rule–based regulatory and metabolic model for Quorum sensing in P. aeruginosa
Source: BMC Syst Biol. 2013 Aug 21;7:81. doi: 10.1186/1752-0509-7-81 (PMC3765737; doi:10.1186/1752-0509-7-81)
Supplement: Additional file 3 — Table S3 Multi–level nodes. Multi–level nodes with their corresponding maximal possible states. [file 1752-0509-7-81-S3.pdf]

node	RsaL	LasB	pyocyanin	Rhm2	HHQ_in	PQS_in	AI–1_in	AI–2_in	HHQ_out	PQS_out	AI–1_out	AI–2_out
state	3	6	6	6	6	6	6	6	∞	∞	∞	∞
