# Supplementary figures and images for: Rule–based regulatory and metabolic model for Quorum sensing in P. aeruginosa
Source: BMC Syst Biol. 2013 Aug 21;7:81. doi: 10.1186/1752-0509-7-81 (PMC3765737; doi:10.1186/1752-0509-7-81)

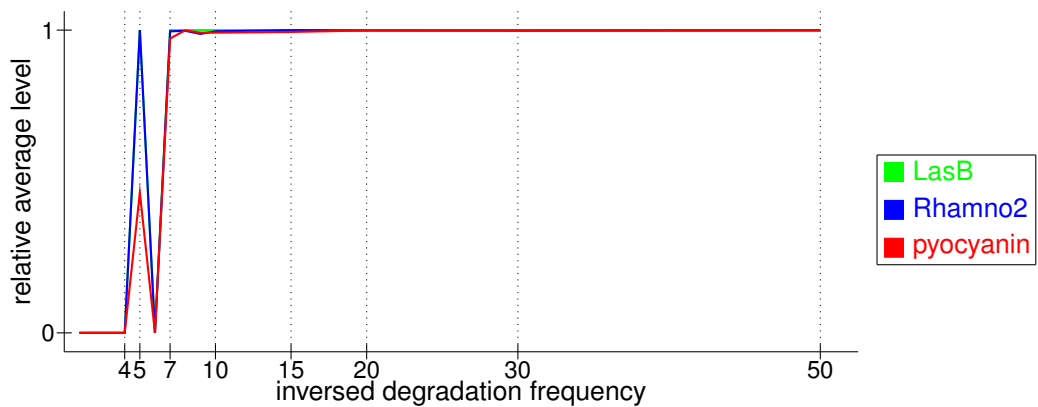

Supplement: Additional file 5 — Figure S1 Influence of degradation frequency. Influence on the concentration levels of the three virulence factors. Relative average values are reported in the time interval 100 to 600 averaged over ten runs obtained with different random numbers. Concentrations of 1 denote values equal to the theoretical maximum value. We used a conversion frequency of 55% for converting HHQ into PQS and a transport threshold of three. [file 1752-0509-7-81-S5.pdf]

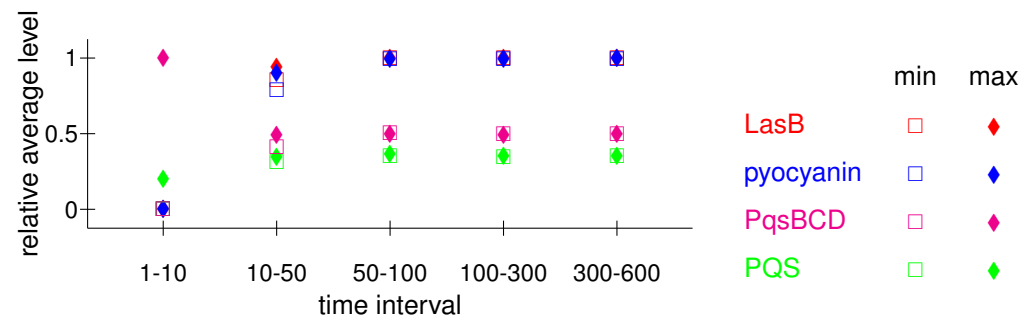

Supplement: Additional file 7 — Figure S3 Influence of different initializations. Influence on LasB, pyocyanin, PqsBCD, and PQS: average values in different time intervals relative to theoretical maximum values averaged over ten runs with different random numbers. In the case of min, a minimal set of nodes (Vfr, C1:G1, C3:G3, and C5:G3) is initially activated, while max means that all nodes (except for LasB, Rhm2, pyocyanin, and external autoinducers) were set to one in the beginning. [file 1752-0509-7-81-S7.pdf]
