# Supplementary material for: Rule–based regulatory and metabolic model for Quorum sensing in P. aeruginosa
Source: BMC Syst Biol. 2013 Aug 21;7:81. doi: 10.1186/1752-0509-7-81 (PMC3765737; doi:10.1186/1752-0509-7-81)
Supplement: Additional file 8 — Table S4 Example trajectory. Level values of nodes in the pqs system in the time interval 10 to 150 considering a wild type cell of the original network with a minimal initial setup. [file 1752-0509-7-81-S8.pdf]

time step	HHQ	PQS	C3	C5	C3:G3	C5:G3	PqsA	PqsBCD	PqsE
10	0	0	0	0	1	1	0	0	0
11	1	1	0	0	0	0	1	1	1
12	2	1	0	0	0	0	0	0	0
13	2	1	0	0	0	0	0	0	0
14	2	1	0	0	0	0	0	0	0
15	2	1	0	0	0	0	0	0	0
16	0	2	0	1	0	0	0	0	0
17	0	2	0	0	0	1	0	0	0
18	1	2	0	0	0	0	1	1	1
19	2	1	1	0	0	0	0	0	0
20	0	1	0	1	1	0	0	0	0
21	0	2	0	0	0	1	1	1	1
22	2	1	1	0	0	0	1	1	1
23	2	1	0	1	0	0	0	0	0
24	0	2	0	1	0	1	0	0	0
25	1	1	1	0	0	1	1	1	1
26	2	2	0	0	0	0	1	1	1
27	1	2	1	0	0	0	0	0	0
28	0	2	1	0	1	0	0	0	0
29	0	2	1	0	1	0	1	1	1
30	1	2	1	0	0	0	1	1	1
31	1	2	1	0	0	0	0	0	0
32	0	2	1	0	1	0	0	0	0
33	0	2	1	0	1	0	1	1	1
34	1	2	1	0	0	0	1	1	1
35	2	1	1	0	0	0	0	0	0
36	1	1	0	1	1	0	0	0	0
37	1	2	0	0	0	1	1	1	1
38	2	2	1	0	0	0	1	1	1
39	1	2	1	0	0	0	0	0	0
40	0	1	1	0	1	0	0	0	0
41	0	2	0	0	1	0	1	1	1
42	1	2	1	0	0	0	1	1	1
43	2	1	1	0	0	0	0	0	0
44	1	1	0	1	1	0	0	0	0
45	0	2	0	0	0	1	1	1	1

46	2	2	1	0	0	0	1	1	1
47	1	2	1	0	0	0	0	0	0
48	0	2	1	0	1	0	0	0	0
49	0	2	1	0	1	0	1	1	1
50	1	2	1	0	0	0	1	1	1
51	2	1	1	0	0	0	0	0	0
52	0	2	0	1	1	0	0	0	0
53	0	2	1	0	0	1	1	1	1
54	2	1	1	0	0	0	1	1	1
55	2	1	0	1	0	0	0	0	0
56	0	2	0	1	0	1	0	0	0
57	1	1	1	0	0	1	1	1	1
58	2	1	0	0	0	0	1	1	1
59	3	1	0	1	0	0	0	0	0
60	1	1	0	1	0	1	0	0	0
61	1	2	0	0	0	1	1	1	1
62	2	1	1	0	0	0	1	1	1
63	3	1	0	1	0	0	0	0	0
64	1	2	0	1	0	1	0	0	0
65	1	2	1	0	0	1	1	1	1
66	2	2	1	0	0	0	1	1	1
67	1	2	1	0	0	0	0	0	0
68	0	2	1	0	1	0	0	0	0
69	0	2	1	0	1	0	1	1	1
70	1	2	1	0	0	0	1	1	1
71	2	1	1	0	0	0	0	0	0
72	0	2	0	1	1	0	0	0	0
73	0	2	1	0	0	1	1	1	1
74	2	1	1	0	0	0	1	1	1
75	2	1	0	1	0	0	0	0	0
76	1	1	0	1	0	1	0	0	0
77	1	2	0	0	0	1	1	1	1
78	2	1	1	0	0	0	1	1	1
79	2	1	0	1	0	0	0	0	0
80	0	1	0	1	0	1	0	0	0
81	1	1	0	0	0	1	1	1	1
82	2	2	0	0	0	0	1	1	1

83	1	2	1	0	0	0	0	0	0
84	1	1	1	0	1	0	0	0	0
85	0	2	0	0	1	0	1	1	1
86	1	3	1	0	0	0	1	1	1
87	1	2	1	0	0	0	0	0	0
88	0	3	1	0	1	0	0	0	0
89	0	2	1	0	1	0	1	1	1
90	1	3	1	0	0	0	1	1	1
91	2	2	1	0	0	0	0	0	0
92	1	1	1	0	1	0	0	0	0
93	0	2	0	0	1	0	1	1	1
94	1	2	1	0	0	0	1	1	1
95	1	2	1	0	0	0	0	0	0
96	1	1	1	0	1	0	0	0	0
97	1	2	0	0	1	0	1	1	1
98	1	2	1	0	0	0	1	1	1
99	1	3	1	0	0	0	0	0	0
100	0	2	1	0	1	0	0	0	0
101	0	2	1	0	1	0	1	1	1
102	1	2	1	0	0	0	1	1	1
103	1	2	1	0	0	0	0	0	0
104	0	2	1	0	1	0	0	0	0
105	0	2	1	0	1	0	1	1	1
106	1	2	1	0	0	0	1	1	1
107	1	2	1	0	0	0	0	0	0
108	0	2	1	0	1	0	0	0	0
109	0	2	1	0	1	0	1	1	1
110	1	2	1	0	0	0	1	1	1
111	1	2	1	0	0	0	0	0	0
112	0	2	1	0	1	0	0	0	0
113	0	3	1	0	1	0	1	1	1
114	1	2	1	0	0	0	1	1	1
115	2	2	1	0	0	0	0	0	0
116	1	1	1	0	1	0	0	0	0
117	0	2	0	0	1	0	1	1	1
118	1	3	1	0	0	0	1	1	1
119	1	2	1	0	0	0	0	0	0

120	0	1	1	0	1	0	0	0	0
121	0	2	0	0	1	0	1	1	1
122	1	3	1	0	0	0	1	1	1
123	1	2	1	0	0	0	0	0	0
124	0	2	1	0	1	0	0	0	0
125	0	2	1	0	1	0	1	1	1
126	1	2	1	0	0	0	1	1	1
127	1	2	1	0	0	0	0	0	0
128	1	1	1	0	1	0	0	0	0
129	0	2	0	0	1	0	1	1	1
130	1	2	1	0	0	0	1	1	1
131	1	2	1	0	0	0	0	0	0
132	1	1	1	0	1	0	0	0	0
133	0	2	0	0	1	0	1	1	1
134	1	2	1	0	0	0	1	1	1
135	2	1	1	0	0	0	0	0	0
136	1	1	0	1	1	0	0	0	0
137	0	2	0	0	0	1	1	1	1
138	2	1	1	0	0	0	1	1	1
139	1	2	0	1	0	0	0	0	0
140	1	0	1	0	0	1	0	0	0
141	2	0	0	0	1	0	1	1	1
142	1	2	0	1	0	0	1	1	1
143	3	1	1	0	0	0	0	0	0
144	1	2	0	1	1	0	0	0	0
145	1	2	1	0	0	1	1	1	1
146	2	2	1	0	0	0	1	1	1
147	1	2	1	0	0	0	0	0	0
148	1	1	1	0	1	0	0	0	0
149	0	2	0	0	1	0	1	1	1
150	1	2	1	0	0	0	1	1	1
