# Supplementary material for: Rule–based regulatory and metabolic model for Quorum sensing in P. aeruginosa
Source: BMC Syst Biol. 2013 Aug 21;7:81. doi: 10.1186/1752-0509-7-81 (PMC3765737; doi:10.1186/1752-0509-7-81)
Supplement: Additional file 4 — Equations. Explicit form of functions required in equation (1). [file 1752-0509-7-81-S4.pdf]

$$K(x,\epsilon) = \begin{cases} 1 & \text{if } x \geq \epsilon \\ 0 & \text{else} \end{cases}$$

$$H(x) = \begin{cases} \lfloor x \rfloor & \text{if } x \geq 0 \\ 0 & \text{else} \end{cases}$$

$$G(x,i) = \begin{cases} x & \text{if maximal possible level of node } i > 1 \\ 0 & \text{else} \end{cases}$$

$$M(x,i) = \begin{cases} x & \text{if maximal possible level of node } i \geq x \\ \text{maximal possible level of node } i & \text{else} \end{cases}$$
