# Supplementary material for: Rule–based regulatory and metabolic model for Quorum sensing in P. aeruginosa
Source: BMC Syst Biol. 2013 Aug 21;7:81. doi: 10.1186/1752-0509-7-81 (PMC3765737; doi:10.1186/1752-0509-7-81)
Supplement: Additional file 6 — Figure S2 Effect of different transport thresholds on elastase production. Starting behavior of LasB comparing a threshold of one (box) and six (square). [file 1752-0509-7-81-S6.pdf]

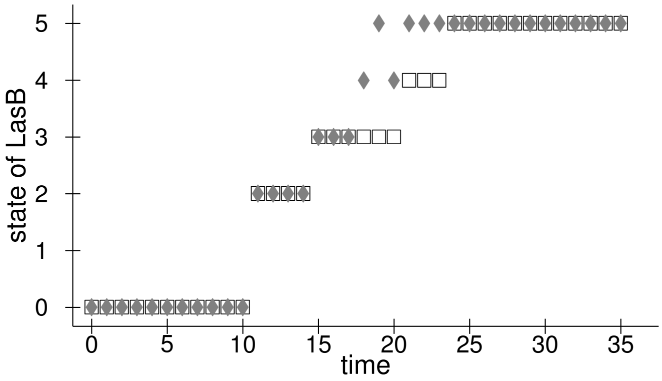
state of LasB
time
0
1
2
3
4
5
0
5
10
15
20
25
30
35
